# Real-World Clinical Characterisation of Polycythaemia Vera Patients from a Prospective Registry in Portugal: Is Resistance to Hydroxyurea a Reality?

**DOI:** 10.3390/hematolrep15030056

**Published:** 2023-09-13

**Authors:** Maria Sarmento, Marta Duarte, Sandra Ponte, Juan Sanchez, Diana Roriz, Laura Fernandes, Maria José Monteiro Silva, Judite Pacheco, Gisela Ferreira, Jorge Freitas, Inês Costa, Daniel Brás

**Affiliations:** 1 Unidade Local de Saúde de Matosinhos, Hospital Pedro Hispano, 4464-513 Senhora da Hora, Portugal; maria.sarmento@ulsm.min-saude.pt (M.S.); marta.duarte@ulsm.min-saude.pt (M.D.); 2 Centro Hospitalar Lisboa Ocidental, Hospital de São Francisco Xavier, 1449-005 Lisbon, Portugal; jsanchez@chlo.min-saude.pt; 3 Fundação Champalimaud, 1400-038 Lisbon, Portugal; diana.roriz@fundacaochampalimaud.pt (D.R.); laura.fernandes@fundacaochampalimaud.pt (L.F.); 4 Centro Hospitalar de Vila Nova de Gaia/Espinho, 4434-502 Vila Nova de Gaia, Portugal; maria.monteiro.silva@chvng.min-saude.pt (M.J.M.S.); judite.pacheco@chvng.min-saude.pt (J.P.); 5 Centro Hospitalar do Baixo Vouga, 3810-164 Aveiro, Portugal; gisela.ferreira.71182@chbv.min-saude.pt; 6 Instituto Português de Oncologia do Porto, 4200-072 Oporto, Portugal; jfreitas@ipoporto.min-saude.pt; 7 Novartis Farma, Produtos Farmacêuticos S.A., 2740-257 Porto Salvo, Portugal; ines-1.costa@novartis.com (I.C.); daniel.bras@novartis.com (D.B.)

**Keywords:** disease management, haemorrhagic events, hydroxyurea, resistance, symptomatic evaluation, patient-reported outcomes, thrombotic events, polycythaemia vera

## Abstract

Patients with polycythaemia vera (PV) are at increased risk of thrombosis and haemorrhages. Although hydroxyurea (HU) has been the frontline therapy for patients at high risk of vascular complications, about 25% of patients develop resistance/intolerance to this therapy. The aim of this non-interventional, multicentre cohort study was to understand the clinical characteristics and HU treatment response of Portuguese PV patients. HU resistance/intolerance was defined according to adjusted European LeukemiaNet (ELN) criteria. In total, 134 PV patients with a mean (SD) disease duration of 4.8 (5.0) years were included and followed up for 2 years. At baseline, most patients were ≥60 years old (83.2%), at high risk for thrombotic events (87.2%), and receiving HU therapy (79.1%). A total of 10 thrombotic events and 8 haemorrhagic events were reported, resulting in a 5-year probability of thrombo-haemorrhagic events of 17.2%. Haematocrit (*p* = 0.007), haemoglobin (*p* = 0.012) and MPN10 symptom score (12.0 (11.6) vs. 10.3 (9.1); *p* = 0.041) decreased significantly at the 24-month visit compared to baseline. Overall, 75.9% of patients met at least one of the adjusted ELN criteria for HU resistance, and 14.4% of patients remained on HU throughout the study. The results from this real-world study may help identify the subset of patients at higher risk for disease sequelae who may benefit from earlier second-line treatment.

## 1. Introduction

Polycythaemia vera (PV) is a chronic myeloproliferative neoplasm (MPN) characterised by a somatic activating mutation in the JAK2 gene that drives abnormal erythrocytosis, resulting in an abnormal increase in red blood cell mass [1,2]. The incidence of PV is around 2 per 100,000 per year, which results in an overall prevalence of 44–57 per 100,000 individuals [3,4].

Patients with PV have an increased risk of thrombosis and haemorrhages, with an associated risk of disease progression to myelofibrosis (MF) or acute myeloid leukaemia (AML) [4,5]. Such clinical outcomes reduce patients’ quality of life and increase their morbidity and mortality. Thus, symptomatic relief and prevention of thrombotic and haemorrhagic events represent primary goals in PV treatment [6,7].

The risk of thrombosis in PV patients is associated with the patients’ age and the likelihood of recurrent thrombosis. Accordingly, PV patients can be stratified into high-risk (age > 60 years or thrombosis history) or low-risk (absence of both risk factors) [8]. The risk of thrombotic events is reduced by maintaining a haematocrit level below 45% [9]. Typical management is accomplished with cytoreductive therapy in high-risk or selected patients and with phlebotomy and low-dose aspirin in all PV patients [6,10].

The first-line cytoreductive therapy of choice is hydroxyurea (HU), which may still require the concomitant use of phlebotomy [11]. However, a Spanish study reported that 12% and 13% of patients developed resistance and intolerance to HU, respectively. HU was also associated with an increased risk of death or haematological transformation [12]. Therefore, clinicians must monitor the response to HU and its side effects in order to decide whether the patient should be switched to second-line therapy.

Clinical characterisation of Portuguese PV patients is scarce. So, the main goal of this study was to characterise the clinical profile, symptomatic burden, and treatment response of Portuguese PV patients. A better understanding of the clinical characteristics of these patients, especially those with an inadequate response or intolerance to HU, is of paramount importance for an optimal treatment approach and the best possible clinical outcomes. 

## 2. Materials and Methods

### 2.1. Study Design and Population

This is a descriptive, non-interventional, prospective, and multicentre cohort study of adult Portuguese patients diagnosed with PV and registered in the Portuguese Oncology Nursing Association (AEOP) database, which includes patients with all Philadelphia-negative myeloproliferative neoplasms (PV, MF, and ET) from 8 participating centres that geographically represent the country. This database was initially established for the assessment and monitoring of symptoms in MPN patients using the MPN-Symptom Assessment Form Total Symptom Score (MNP-10) [13]. With the exception of the MNP10 scale, all clinical information was collected by healthcare professionals from patients’ medical records as part of routine care (secondary use of data). The AEOP database index date was 21 March 2017. This corresponded to the first patient entry and assessment of symptom burden using the MNP10 questionnaire. 

The study period was one year (1 November 2020 to 20 October 2021), during which patient data were collected from the first registry up to a maximum of 24 months. The patient’s first registry into the database was defined as the baseline visit, and the data collected at 12 and 24 months after study initiation were considered the two follow-up visits. The observation period spanned from the index date until death, loss to follow-up, or the end of the study at month 24. 

Adult patients (>18 years) with a confirmed diagnosis of PV according to the hospital medical record (patient diary) and registered in the AEOP database were included in the study.

### 2.2. Study Endpoints

The characterisation of PV patients’ demographics, clinical profile, treatment patterns and symptom burden (assessed through the MNP10 score) were considered the primary endpoints.

The secondary endpoints included the description of thrombotic and haemorrhagic events, the proportion of patients with resistance/intolerance to HU and the assessment of the association between potential risk factors and key clinical outcomes. 

The evaluation of resistance/intolerance to HU was performed based on adjusted ELN criteria [14,15], which were adapted to this study’s characteristics. These criteria were applied to all patients on any HU dosage for at least 9 months, instead of considering the maximum tolerated HU dose. Resistance was defined in patients meeting at least one of the following criteria: need for phlebotomy, platelet count > 400 × 10^9^/L, leukocyte count > 10 × 10^9^/L and neutrophil count < 1.0 × 10^9^/L, or platelet count < 100 × 10^9^/L, or haemoglobin < 10.0 g/dL. Symptoms were evaluated through the MPN10 score and considered when the MPN10 total score ≥ 20 or an individual symptom score > 5 [16]. 

Intolerance was considered whenever any unacceptable HU-related non-haematological toxicities occurred. 

### 2.3. Data Collection

Demographic data (age and sex) were collected at baseline. Clinical data (disease duration, therapy, haematocrit level, platelet count, leukocyte count, haemoglobin level and neutrophil count) were collected at baseline and at the 12- and 24-month follow-up visits. 

As recommended by the NCCN guidelines, PV patients’ symptoms were assessed and monitored using the MPN-SAF total symptom score, also known as the MPN10 scale [17]. During the course of their treatment, patients self-evaluated 10 of the most clinically significant symptoms, with a total score ranging from 0 to 100 points [13]. 

Clinician-reported cases of MF progression, AML transformation, non-haematological toxicities, and resistance/intolerance to HU were also recorded.

### 2.4. Statistical Analysis

Categorical variables were summarised by providing absolute and relative frequencies. Continuous variables were summarised considering the mean, standard deviation (SD), median, and 25th and 75th percentiles (P25 and P75). 

The normality of the distribution of continuous variables was assessed using histograms, Q-Q plots, and the Shapiro–Wilk test. The comparison of paired observations, from baseline to 12 and 24 months, for continuous variables was performed using the non-parametric Wilcoxon test for paired samples. The Bonferroni method was used to adjust the significance level for the multiple comparisons. 

For the symptom scale, the significance level was adjusted when comparing the results of each symptom. A Kaplan–Meier analysis was performed to estimate the time to thrombotic and haemorrhagic events since PV diagnosis (maximum registered time of 240 months). 

A significance level of 5% was considered. Two-sided 95% confidence intervals (CIs) for the mean and for percentages were provided for the main results. Statistical analysis was conducted using the R^®^ software version 4.1.2.

## 3. Results

### 3.1. Demographic and Clinical Profile of PV Patients

A total of 134 PV patients registered in the AEOP database were included at baseline; 12-month follow-up data was available for 65 patients, and 24-month follow-up data was available for 37 patients. 

At baseline, patients had a mean (SD) age of 70.6 (11.7) years, with the majority being female (71, 53%). Still, a considerable number (22, 16.8%) of patients < 60 years old were included. The mean (SD) disease duration was 4.8 (5.0) years, with a total of 21 (17.2%) patients having a PV diagnosis for ≥10 years. Based on the classical risk stratification for thrombotic events in PV patients (age > 60 years or thrombotic history), 104 participants (83.2%) were considered high-risk PV patients at the time of study initiation (Table 1). No differences were observed between the group of patients classified as low-risk and those classified as high-risk with respect to mean disease duration (5.2 years vs. 4.7 years, respectively; *p* = 0.524), nor with respect to the female-to-male ratio between the groups (*p* = 0.157) (Table S1).

### 3.2. Treatment Characteristics

At enrolment, the majority of patients (*n* = 106, 79.1%) were already receiving pharmacological therapy (Table 2), with HU being the most frequent treatment (106, 79.1%), administered for a median (P25–P75) of 1.9 (0.2–3.4) years. HU was taken either alone (66, 49.2%) or in combination with other drugs (40, 29.8%), mainly acetylsalicylic acid (ASA; 38, 28.4%). Phlebotomy was added to HU therapy in 35 patients (26.1%). Pharmacological treatment options other than HU were ASA alone (6, 4.5%), ASA with interferon (1, 0.7%), ruxolitinib (5, 3.7%), or busulfan (1, 0.7%). Of note, the percentage of individuals receiving HU remained relatively consistent across all follow-up visits, ranging from 79.1% at baseline to 75.7% at the 24-month follow-up visit. The same tendency was observed regarding the use of other medications. The percentage of patients who did not receive any pharmacological treatment ranged from 10.8% to 12.3% throughout the study.

In high-risk patients, HU was the cytoreductive therapy of choice (89, 81.6%), combined or not with other modalities (phlebotomy: 29, 26.6%; ASA: 30, 27.5%; warfarin: 1, 0.9%; clopidogrel: 1, 0.9%). Four (3.7%) of the high-risk patients received ruxolitinib; another four took ASA; and one (0.9%) was on busulfan. Eleven (10.1%) patients had no pharmacological treatment, of whom three (2.8%) received phlebotomy.

Of the 81 participants who had information on the number of phlebotomies performed prior to study initiation, 44 (54.3%) had a record of having undergone this procedure. Twelve patients (30.0%) had between one and two phlebotomies during the first 12 months of follow-up. In the second year, this number was reduced to eight (25.8%) patients. A total of four (10.0%) and three (9.7%) PV patients received three or more phlebotomies during the first and second year of follow-up, respectively. The percentage of individuals who did not receive phlebotomy increased from 45.7% at baseline to 74.2% at 24 months. Of note is that a substantial number of patients had missing data regarding phlebotomy (Table 2). 

### 3.3. Clinical Outcomes and Haematological Response

**Figure 1:**
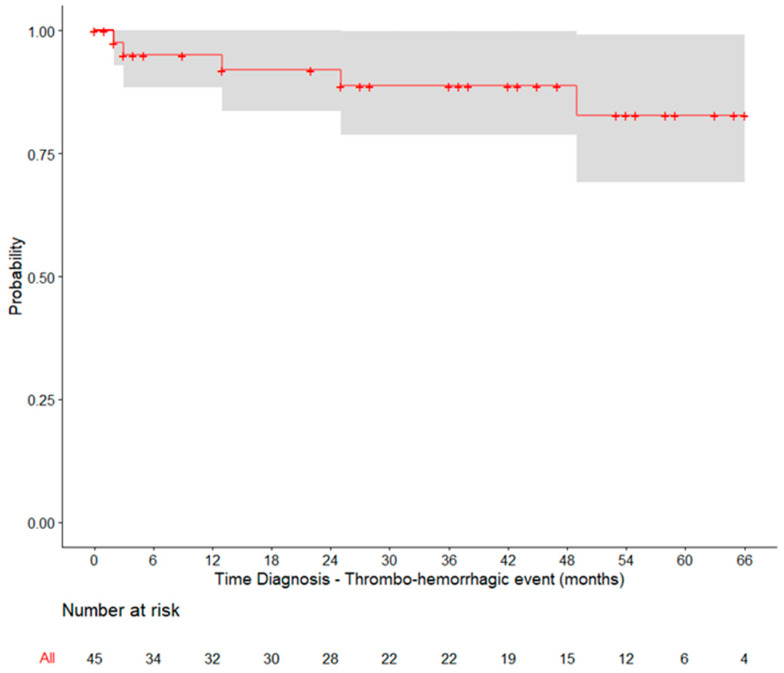
Kaplan–Meier curve for the time between PV diagnosis and the occurrence of thrombo-haemorrhagic events. Only patients diagnosed with PV within up to 5 years of the index date were included.

Over a median of 4.9 years since PV diagnosis, nine (6.9%) patients reported a total of ten thrombotic events (3 arterial and 7 venous) and five (6.0%) patients reported a total of eight events (Table S2). This resulted in 1-, 2-, and 5-year probabilities of thrombo-haemorrhagic events of 5.0%, 8.0% and 17.2%, respectively, when considering data from the first 5 years after PV diagnosis (Figure 1, Table S3).

Throughout the study, clinicians reported two cases of progression to MF in patients whose PV was diagnosed 16.5 years and 25 months prior to baseline. AML transformation was not reported. Non-haematological toxicity was recorded in two patients, namely tinnitus and bradycardia (Table S4). The patient with bradycardia was on HU treatment throughout the entire study period.

An analysis of the haematological data (Table 3) revealed significant differences between the baseline and 24-month mean values for haematocrit and haemoglobin (*p* = 0.007 and *p* = 0.012, respectively). Specifically, the mean (SD) haematocrit percentage decreased from 45.2 (6.8) at baseline to 42.2 (5.2) at 24 months. For haemoglobin, the mean value decreased from 14.6 (2.6) at baseline to 13.5 (1.8) at 24 months. 

In addition to these parameters, the data suggest that patients in this cohort experienced a slight decrease in the platelet and neutrophil counts during the 24 months of follow-up, while their white blood cell counts remained relatively stable. 

### 3.4. Resistance to HU

The evaluation of resistance to HU was based on the modified ELN criteria [14,15], adapted to the conditions of this study. The modified ELN criteria consider resistance to occur when there is the need for phlebotomy, uncontrolled myeloproliferation, or failure to reduce splenomegaly in patients treated with 2 g of HU per day for at least 3 months or the maximum tolerated dose, or the occurrence of cytopaenia at the lowest dose. In addition, according to the same criteria, intolerance to HU is recognised in the case of unacceptable HU-related non-haematological toxicity. However, in this study, data on the maximum tolerated dose of HU were not recorded, and non-haematological toxicities were only reported in about half the patients. Therefore, resistance to HU was analysed cautiously in patients receiving HU for at least 9 months while using the MPN10 scale to assess symptomatic burden. HU intolerance could not be determined.

Overall, 83 patients had been treated with HU for at least 9 months. Of these, 63 patients (75.9%) met at least one ELN criterion, defining resistance to HU. At the time of study enrolment, 45 (76.3%) of the 59 patients treated with HU for ≥9 months met at least one of these criteria: 9 patients required phlebotomy, 4 had elevated platelet and leukocyte counts, 18 developed cytopaenia, and 37 had an MPN10 score ≥ 20 or an individual symptom score of >5. Information on splenomegaly was not available for 86% of the patients. At the 24-month follow-up, 15 (62.5%) of the 24 patients on HU for ≥9 months met the same criteria (Table 4). Among patients receiving HU for ≥9 months, nine (15.3%) maintained resistance to HU from baseline to the end of the study period, in agreement with the adjusted ELN criteria (Table S5). At the 12-month follow-up, six of these patients (66.7%) remained on the initial HU dose. No HU dose-related information was available for the remaining three patients.

The majority of patients relied solely on HU treatment for haematocrit control at baseline, although a substantial number of individuals also received phlebotomy either in combination with HU or as a standalone approach. Specifically, among the 46 patients who had controlled haematocrit levels (<45%) at baseline, the treatment patterns were as follows: 3 patients (6.5%) received only phlebotomy before or up to the baseline; 12 patients (26.1%) underwent both phlebotomy and hydroxyurea (HU) treatment; 25 patients (54.3%) received HU treatment alone; and 6 patients (13.0%) did not receive HU or phlebotomy (Table S6).

The analysis of patients with controlled haematocrit levels at 24 months (*n* = 20) revealed variations in treatment patterns over time, with some patients transitioning from or combining HU with phlebotomy. 

The number of phlebotomies performed before and after HU initiation is shown in Table 5. Of note, only patients with at least 12 months of follow-up (and up to 24 months) were included in the analysis. Prior to the initiation of the cytoreductive therapy, only eight patients (14.3%) had received phlebotomy. However, following the initiation of HU treatment, there was a significant increase (*p* = 0.005) in the number of patients requiring phlebotomy for disease control, with a total of 17 patients undergoing the procedure at least once (30.7% vs. 14.3% before HU).

### 3.5. Patient-Reported Symptom Burden

Self-evaluation of the ten most clinically significant symptoms was conducted using the MPN10 scale (Table 6). At baseline, the mean (SD) MPN10 total score was 12.0 (11.6). Among the reported symptoms, fatigue, itching and inactivity exhibited the highest burden. Moreover, these three symptoms consistently remained the heaviest throughout the study, without statistically significant alterations compared to baseline. Yet, there was a significant decrease in the total MPN10 score after 24 months of study initiation (*p* = 0.041).

## 4. Discussion

This real-world study provides an extensive clinical characterisation of Portuguese PV patients, enabling a better understanding of the patient demographics, risk profiles, symptomatology, treatment patterns, and clinical outcomes associated with this condition. This knowledge has the potential to inform clinical decision making, improve patient management strategies, and facilitate the development of personalised and more effective therapeutic interventions for PV patients in the future.

The baseline characteristics of the study cohort align with previous observations in PV patients, with the majority of patients being older than 60 years and classified as high-risk based on age or thrombotic history. These factors are known to increase the risk of thrombotic events in these patients [18], underscoring the urgent need for effective treatment management strategies to mitigate these complications.

Hydroxyurea (HU) was the frontline therapy for the majority of patients at the time of study initiation, consistent with its recommendation as the first-line cytoreductive therapy for high-risk PV patients. However, the challenges associated with its inaccurate management should not be overlooked [8,12]. Resistance and intolerance to this drug can reach significant numbers, with HU resistance posing a heightened risk of mortality and disease transformation to acute leukaemia or MF [12]. Indeed, a significant number of patients met at least one of the adjusted ELN criteria defining HU resistance, with 14% of patients maintaining HU resistance throughout the study period without discontinuing the treatment. These results are consistent with previous clinical experience indicating that a large proportion of patients (20–60%) remain on HU therapy despite a lack of response or intolerance [19]. The prevalence of resistance to HU has been reported in various studies, ranging from 11% to 21% of patients [12,15,17,20]. Our study found a similar rate of 15% for patients receiving HU for ≥9 months and maintaining at least one criterion for HU resistance from baseline to the end of the study period. Unfortunately, the assessment of HU intolerance was not possible due to a lack of information on non-hematologic toxicities. Taken together, these findings highlight the lack of standard therapeutic approaches and the need to monitor treatment response and consider second-line therapies for non-responders.

The analysis of treatment patterns in the population with controlled haematocrit levels at baseline showed that while the majority of patients received HU alone, a substantial proportion also received phlebotomy, either in combination with HU or as a stand-alone approach. However, the analysis of patients with haematocrit levels < 45% at 24 months revealed variations in treatment patterns over time, with some patients switching from HU to other interventions or combining HU with phlebotomy. In addition, a significant increase in the number of patients requiring phlebotomy for disease control after the initiation of HU treatment was observed, further supporting the poor management of these patients in routine practice. 

The thrombotic and haemorrhagic events reported in this study illustrate the ongoing risk faced by Portugese PV patients, despite treatment. The projected 5-year probability of thrombo-haemorrhagic events was 17.2%, similar to previous studies [7,17]. The fact that approximately one-sixth of the study sample was under the age of 60, and more than half was under the age of 74, underscores the importance of maintaining ongoing vigilance and implementing appropriate management strategies to prevent such complications, especially in patients with a relatively long life expectancy.

The MPN10 scale, which assesses the ten most clinically important symptoms experienced by PV patients, was used to assess symptom burden. Fatigue, itching and inactivity were the most burdensome symptoms reported at baseline, and their severity remained relatively unchanged throughout the study. Even though the observed total MPN10 scores were relatively lower than those reported for other cohorts (mean, SD: 18.8, 15.5) [21], fatigue, itching and inactivity were the symptoms with the highest burden, consistent with previous studies [21]. The overall decrease in the total MPN10 score at 24 months suggests a potential improvement in symptom burden, although the clinical significance of this finding should be interpreted with caution.

Furthermore, PV patients may experience HU-related non-haematological toxicity and symptoms that must be recognised and adjusted to fit the therapeutic plan. In this cohort, one patient developed tinnitus and another bradycardia. Still, only the latter was on HU treatment.

A major limitation of this study pertained to the use of secondary data. Patient information was occasionally incomplete, particularly their maximum tolerated HU dose, which was not documented in this study, and there were missing data for certain evaluated criteria. These limitations affected the assessment of predictive factors for thrombotic and haemorrhagic events and other clinical outcomes in this Portuguese cohort. Moreover, they hindered the accurate determination of the frequency of patients who developed resistance to HU, which was cautiously focused on patients who had been treated with HU for at least 9 months and based on the MPN10 symptom scale. 

## 5. Conclusions

This real-world study provides the first clinical characterisation of Portuguese PV patients. The results show that a significant proportion of PV patients meet HU resistance criteria, with an alarming number of HU-treated patients maintaining resistance throughout the study without treatment change. The lack of standardised management of PV highlights the importance of establishing a consensus on optimal treatment strategies. This also has an impact on the quality of life of these patients, as evidenced by their symptom burden. Overall, this study underlines the importance of identifying patients with HU resistance and implementing a standardised approach to ensure the optimal treatment and management of PV, ultimately improving patient clinical outcomes.

## Figures and Tables

**Table 1:** Characteristics of PV patients at baseline.

Baseline Characteristics	Overall (*n* = 134)
**Age, years**	*n* = 131
18–34	2 (1.5%)
35–59	20 (15.3%)
60–74	55 (42.0%)
≥75	54 (41.2%)
Mean [95% CI], (SD)	70.6 [69.0, 72.2], (11.7)
Missing	3 (2.2%)
**Sex**	*n* = 134
Male	63 (47.0%)
Female	71 (53.0%)
**Disease duration, years**	*n* = 122
<1 year	33 (27.0%)
1–4 years	40 (32.8%)
5–9 years	28 (23.0%)
10–14 years	11 (9.0%)
≥15 years	10 (8.2%)
Mean [95% CI], (SD)	4.8 [4.1, 5.5], (5.0)
Missing	12 (9.0%)
**PV risk status**	*n* = 125
Low	21 (16.8%)
High	104 (83.2%)
Missing	9 (6.7%)
**Thrombotic event history**	*n* = 78
Prior thrombotic event	5 (6.4%)

**Table 2:** Treatment profile of PV patients.

Treatment Profile	Baseline	12 Months	24 Months
**Pharmacological treatment**	*n* = 134	*n* = 65	*n* = 37
HU	106 (79.1%)	52 (80.0%)	28 (75.7%)
	[72.2%, 86.0%]	[70.3%, 89.7%]	[61.9%, 89.5%]
Alone	66 (49.2%)	33 (50.8%)	18 (48.6%)
With acetylsalicylic acid (ASA)	38 (28.4%)	17 (26.2%)	8 (21.6%)
With clopidogrel	1 (0.7%)	1 (1.5%)	1 (2.7%)
With warfarin	1 (0.7%)	1 (1.5%)	0 (0.0%)
With triflusal	0 (0.0%)	0 (0.0%)	1 (2.7%)
With phlebotomy	35 (26.1%)	15 (23.1%)	11 (29.7%)
Other	13 (9.7%)	5 (7.7%)	4 (10.8%)
Without pharmacological treatment	15 (11.2%)	8 (12.3%)	4 (10.8%)
**Phlebotomy**	*n* = 81	*n* = 40	*n* = 31
No	37 (45.7%)	24 (60.0%)	23 (74.2%)
	[34.8%, 56.5%]	[44.8%, 75.2%]	[58.8%, 89.6%]
Yes	44 (54.3%)	16 (40.0%)	8 (25.8%)
	[43.5%, 65.1%]	[24.8%, 55.2%]	[10.4%, 41.2%]
Number of phlebotomies			
1–2	30 (37.0%)	12 (30.0%)	5 (16.1%)
	[26.5%, 47.5%]	[15.8%, 44.2%]	[3.2%, 29%]
≥3	10 (12.3%)	4 (10.0%)	3 (9.7%)
	[5.1%, 19.5%]	[0.7%, 19.3%]	[0.0%, 20.1%]
Unknown	4 (4.9%)	0 (0.0%)	0 (0.0%)
	[0.2%, 9.6%]		
Missing	53 (39.6%)	25 (38.5%)	6 (16.2%)

**Table 3:** Haematological data of PV patients throughout the study.

	Baseline	12 Months	*p*-Value ^1^	24 Months	*p*-Value ^2^
**Haematocrit (%)**	*n* = 100	*n* = 44 ^1^		*n* = 31 ^2^	
Mean (SD)	45.2 (6.8)	43.7 (4.2)	0.056	42.2 (5.2)	0.007
Median	45.5	43.5		43.0	
**Haemoglobin (g/dL)**	*n* = 118	*n* = 44 ^3^		*n* = 31 ^4^	
Mean (SD)	14.6 (2.0)	14.2 (1.5)	0.202	13.5 (1.8)	0.012
Median	14.7	14.4		13.6	
**Platelets (×10^9^/L)**	*n* = 100	*n* = 45 ^5^		*n* = 31 ^6^	
Mean (SD)	350.2 (194.2)	303.6 (131.5)	0.492	325.2 (130.8)	0.493
Median	298.0	260.0		327.0	
**Neutrophils (×10^9^/L)**	*n* = 29	*n* = 16 ^7^		*n* = 13 ^8^	
Mean (SD)	6.69 (4.14)	6.61 (5.34)	0.847	5.37 (3.31)	0.108
Median	5.2	4.9		3.7	
**Leukocytes (×10^9^/L)**	*n* = 72	*n* = 34 ^9^		*n* = 30 ^10^	
Mean (SD)	8.8 (4.2)	9.2 (5.2)	0.424	9.2 (5.4)	0.814
Median	7.5	7.0		7.4	

Multiple comparisons of hematological data between baseline and each follow-up visit were performed considering Wilcoxon test for paired samples and a significance level adjusted by the Bonferroni method: 0.050/2 = 0.025: ^(1)^ *p* = 0.056; ^(2)^ *p* = 0.007; ^(3)^ *p* = 0.202; ^(4)^ *p* = 0.012; ^(5)^ *p* = 0.492; ^(6)^ *p* = 0.493; ^(7)^ *p* = 0.847; ^(8)^ *p* = 0.108; ^(9)^ *p* = 0.424; ^(10)^ *p* = 0.814.

**Table 4:** Number of PV patients on HU for ≥9 months meeting at least one of the adjusted ELN criteria.

Number of Patients	Baseline (*n* = 134)	12 Months (*n* = 65)	24 Months (*n* = 37)
HU			
No	28 (20.9%)	13 (20.0%)	9 (24.3%)
	[14.9%, 28.5%]	[12.1%, 31.3%]	[13.4%, 40.1%]
Yes	106 (79.1%)	52 (80.0%)	28 (75.7%)
	[71.5%, 85.1%)	[68.7%, 87.9%]	[13.4%, 40,1%]
On HU for <9 months or no info about HU date	47 (44.3%) [35.2%, 53.8%]	3 (5.8%) [2.0%, 15.6%]	4 (14.3%) [5.7%, 31.5%]
On HU for ≥9 months	59 (55.7%)	49 (94.2%)	24 (85.7%)
	[46.2%, 64.8%]	[84.4%, 98.0%]	[68.5%, 94.3%]
Meeting at least one of the adjusted ELN criteria	45 (76.3%) [65.4%, 87.2%]	29 (59.2%) [45.4%, 73.0%]	15 (62.5%) [43.1%, 81.9%]
Meeting no adjusted ELN criteria	5 (8.5%) [1.4%, 15.6%]	12 (24.5%) [12.4%, 36.5%]	0 (0.0%)
No information available	9 (15.3%) [6.1%, 24.5%]	8 (16.3%) [6.0%, 26.6%]	9 (37.5%) [18.1%, 56.9%]

**Table 5:** Number of patients who received phlebotomy before and after starting HU.

Number of Phlebotomies ^1^	Before HU	After HU
0	48 (85.7%)	39 (69.6%)
1	7 (12.5%)	9 (16.1%)
2	0 (0.0%)	2 (3.6%)
3	1 (1.8%)	2 (3.6%)
4	0 (0.0%)	2 (3.6%)
9	0 (0.0%)	1 (1.8%)
17	0 (0.0%)	1 (1.8%)

^1^ Distribution of patients (*n* = 56) in both groups (before and after HU) was compared using the Wilcoxon test for paired samples: *p* = 0.005.

**Table 6:** Self-assessment of the most clinically significant symptoms by PV patients.

Symptoms: Mean (SD)	Baseline (*n* = 123)	24 Months (*n* = 29)	*p*-Value ^1^
Fatigue	3.0 (2.9)	2.0 (2.5)	0.065
Early satiety	0.8 (1.9)	0.4 (0.9)	0.125
Abdominal discomfort	0.8 (1.9)	0.9 (2.0)	0.354
Inactivity	1.7 (2.6)	2.0 (2.6)	0.809
Concentration problems	1.1 (2.4)	1.1 (1.8)	0.844
Night sweats	1.0 (2.0)	0.4 (1.0)	0.195
Itching	2.0 (2.8)	1.7 (1.9)	0.010
Bone pain	1.2 (2.2)	1.4 (2.3)	0.203
Fever (≥37.8 °C)	0.0 (0.1)	0.0 (0.0)	N/A
Weight loss	0.5 (1.6)	0.3 (1.0)	0.182
MPN10 score	12.0 (11.6)	10.3 (9.1)	0.041

^1^ Comparison of scores between baseline and the 24-month follow-up visit was performed considering the Wilcoxon test for paired samples. For the score of each symptom, the *p*-value was adjusted by the Bonferroni method: *p* = 0.005. N/A: not applicable.

## Data Availability

The datasets generated during and/or analysed during the current study are available from the corresponding author on reasonable request.

## Supplementary Materials

The following supporting information can be downloaded at: https://www.mdpi.com/article/10.3390/hematolrep15030056/s1, Table S1: Gender and mean disease duration of low- and high-risk PV patients; Table S2: Types of thrombotic and hemorrhagic events since PV diagnosis; Table S3. Probabilities of thrombo-haemorrhagic events for the first 5 years after PV diagnosis; Table S4: Clinical outcomes of PV; Table S5: Clinical outcomes of PV; Table S6: Treatment profile of patients with hematocrit level < 45%.
