# Supplementary material for: Real-World Clinical Characterisation of Polycythaemia Vera Patients from a Prospective Registry in Portugal: Is Resistance to Hydroxyurea a Reality?
Source: Hematol Rep. 2023 Sep 13;15(3):532–42. doi: 10.3390/hematolrep15030056 (PMC10530755; doi:10.3390/hematolrep15030056)
Supplement: Supplementary file 1 [file hematolrep-15-00056-s001.zip › hematolrep-2446648-supplementary.pdf]

Research Paper

Real-World Clinical Characterisation of Polycythaemia Vera Patients from a Prospective Registry in Portugal: Is Resistance to Hydroxyurea a Reality?

Maria Sarmento[1], Marta Duarte[1], Juan Sanchez[2], Sandra Ponte[2,*], Diana Roriz[3], Laura Fernandes[3], Maria José Monteiro Silva[4], Judite Pacheco[4], Gisela Ferreira[5], Jorge Freitas[6], Inês Costa[7], Daniel Brás[7]

[1]Unidade Local de Saúde de Matosinhos, Hospital Pedro Hispano, Senhora da Hora, Portugal;
[2]Centro Hospitalar Lisboa Ocidental, Hospital de São Francisco Xavier, Lisbon, Portugal; smponte@chlo.min-saude.pt
[3] Fundação Champalimaud, Lisbon, Portugal;
[4] Centro Hospitalar de Vila Nova de Gaia/Espinho, Vila Nova de Gaia, Portugal;
[5] Centro Hospitalar do Baixo Vouga, Aveiro, Portugal;
[6] Instituto Português de Oncologia do Oporto, Porto, Portugal;
[7] Novartis Farma, Produtos Farmacêuticos S.A, Porto Salvo, Portugal;

*Correspondence: smponte@chlo.min-saude.pt

SUPPLEMENTARY INFORMATION

TABLES

Table S1. Gender and mean disease duration of low- and high-risk PV patients.

Baseline characteristics	Low-risk (n=21)	High-risk (n=104)
Gender	n=21	n=104
Male	13 (61.9%)	46 (44.2%)
Female	8 (38.1%)	58 (55.8%)
Disease duration, years	n=20	n=102
<1 year	4 (20.0%)	29 (28.4%)
1 - 4 years	6 (30.0%)	34 (33.3%)
5 - 9 years	7 (35.0%)	21 (20.6%)
10 - 14 years	1 (5.0%)	10 (9.8%)
≥15 years	2 (10.0%)	8 (7.8%)
Mean ± SD	5.2 ± 4.9	4.7 ± 5.1
Missing	1 (4.8%)	2 (1.9%)

Table S2. Types of thrombotic and haemorrhagic events since PV diagnosis

	n=134
Thrombotic events	10
Arterial	
Extensive myocardial ischemia	1
Pulmonary embolism thrombus	1
Retinal vascular thrombosis	1
Venous	
Deep vein thrombosis	2
Portal vein thrombosis	1
Unspecified myocardial infarction venous insufficiency	1
Splenic vein thrombosis	1
Superficial venous thrombosis	1
Thrombosis	1
Haemorrhagic events	8
Stroke	3
Ischaemic infarction	2
Gastric ulcer - towel hemorrhage	1
Upper gastrointestinal bleeding	1
Acute myocardial infarction	1

Table S3. Probabilities of thrombo-haemorrhagic events for the first 5 years after PV diagnosis.

	% [95% CI]
6 months	5.0% [0.0, 11.5]
12 months	5.0% [0.0, 11.5]
24 months	8.0% [0.0, 16.3]
36 months	11.3% [0.1, 21.2]
48 months	11.3% [0.1, 21.2]
60 months	17.2% [0.9, 30.8]

NOTE: only data from the first five years after PV diagnosis was considered.

Table S4. Clinical outcomes of PV

	Baseline	12 months	24 months
MF progression	n=66	n=29	n=22
No	65 (98.5%)	29 (100.0%)	21 (95.5%)
Yes	1 (1.5%)	0 (0.0%)	1 (4.5%)
AML transformation	n=66	n=29	n=22
No	66 (100.0%)	29 (100.0%)	22 (100.0%)
Yes	0 (0.0%)	0 (0.0%)	0 (0.0%)
Non-haematological toxicity	n=66	n=29	n=23
Not available	21 (31.8%)	17 (58.6%)	14 (60.9%)
No	44 (66.7%)	12 (41.4%)	8 (34.8%)
Yes	1 (1.5%)	0 (0.0%)	1 (4.3%)
Tinnitus	1 (1.5%)	0 (0.0%)	0 (0.0%)
Bradycardia	0 (0.0%)	0 (0.0%)	1 (4.3%)

Table S5. Clinical outcomes of PV

Baseline	12 M	24 M
Neutrophils <10<br>Phlebotomy<br>Total symptom score ≥20<br>Individual symptom score >5	Neutrophils <10<br>Individual symptom score >5	Neutrophils <10
Neutrophils <10<br>Phlebotomy<br>Total symptom score ≥20	Phlebotomy	Neutrophils <10
Neutrophils <10	Neutrophils <10<br>Phlebotomy<br>Total symptom score ≥20	Neutrophils <10
Neutrophils <10<br>Total symptom score ≥20<br>2 Individual symptom score >5	Neutrophils <10	Neutrophils <10
Neutrophils <10<br>Phlebotomy<br>Total symptom score ≥20	Phlebotomy<br>Individual symptom score >5	Neutrophils <10<br>Phlebotomy<br>Platelet count >400×10^9/L and leukocyte count >10×10^9/L
Total symptom score ≥20<br>Individual symptom score >5	Phlebotomy	Phlebotomy

Neutrophils <10<br>Total symptom score ≥20<br>2 Individual symptom score >5<br>Platelet count >400×10⁹/L and leukocyte count >10×10⁹/L	2 Individual symptom score >5<br>Platelet count >400×10⁹/L and leukocyte count >10×10⁹/L	Platelet count >400×10⁹/L and leukocyte count >10×10⁹/L
Phlebotomy	Neutrophils <10<br>Phlebotomy<br>2 Individual symptom score >5	Phlebotomy
Neutrophils <10	Platelet count >400×10⁹/L and leukocyte count >10×10⁹/L	Platelet count >400×10⁹/L and leukocyte count >10×10⁹/L

Table S6. Treatment profile of patients with haematocrit level <45%.

**Haematocrit <45%**	Baseline (n=46)	24 months (n=20)
Phlebotomy alone	3 (6.5%)	0 (0.0%)
HU alone	25 (54.3%)	14 (70%)
Phlebotomy + HU	12 (26.1%)	6 (30%)
No phebotomies or HU	6 (13.0%)	0 (0.0%)
